# Risk Analysis of Latent Tuberculosis Infection among Health Workers Compared to Employees in Other Sectors

**DOI:** 10.3390/ijerph17134643

**Published:** 2020-06-28

**Authors:** Lisa Hermes, Jan Felix Kersten, Albert Nienhaus, Anja Schablon

**Affiliations:** 1 Competence Center for Epidemiology and Health Services Research for Healthcare Professionals (CVcare), Institute for Health Services Research in Dermatology and Nursing (IVDP), University Medical Center Hamburg-Eppendorf, 20246 Hamburg, Germany; Lisa.Hermes@evangelischeskrankenhaus.de (L.H.); Albert.Nienhaus@bgw-online.de (A.N.); A.Schablon@uke.de (A.S.); 2 Department of Occupational Medicine, Hazardous Substances and Public Health, Institution for Statutory Accident Insurance and Prevention in the Health and Welfare Services (BGW), 22089 Hamburg, Germany

**Keywords:** occupational disease, tuberculosis, health workers, LTBI, infection

## Abstract

Latent tuberculosis infection (LTBI) represents a work-related risk for health workers (HWs). Tuberculosis remains the second most common occupational infectious disease among HWs in Germany. Comparative figures on LTBI prevalence in the general population are missing because testing is only carried out in the context of an outbreak situation. The objective of this study is to investigate whether HWs are at greater risk of LTBI than workers in other sectors. This study is based on two samples. The first sample is a database of HWs who were examined by the German Occupational Physicians Network using an interferon-gamma release assay (IGRA). The second sample consists of general employees (non-health workers, non-HWs) from Hamburg who had no professional contact with the health care system. Propensity score matching (PS matching) was performed to ensure better comparability of the groups. The differences in the prevalence of positive test results from IGRAs were examined using univariate and multivariate analyses. After the PS matching of 1:10, 100 test subjects in the non-HW group and 1000 HWs remained to form the analysis collective. The HWs tended to exhibit higher IGRA values than non-HWs. The univariate analysis showed an odds ratio (OR) of 3.86 for the HWs (95% confidence interval (CI): 0.99 to 32.5; *p* = 0.056) with respect to a positive test result. The multivariate analysis produced an OR of 4.92, (95% CI: 1.3 to 43.7; *p* = 0.013) for HWs born in Germany. Despite the declining tuberculosis incidence rates in Germany, a comparison with non-exposed professional groups showed that HWs are at greater risk of LTBI. Preventive medical check-ups still seem to be indicated.

## 1. Introduction

Latent tuberculosis infection (LTBI) and active tuberculosis (TB) represent occupational risks in health care [1,2]. The risk of infection among health workers (HWs) has been described in numerous studies [2,3,4,5]. Worldwide, Germany is among the low-incidence nations with an incidence rate of 6.5 cases per 100,000 inhabitants [6]. The reduction in TB incidence should correspond with a decrease in the risk of TB infection for HWs. However, an additional risk from working in health care seems to persist even in high-income countries with high hygiene standards [7,8]. Tuberculosis, as an occupational disease among HWs, is still the second most common recognized occupational disease [9]. TB screening is, therefore, also performed for HWs in low-incidence countries, in order to prevent nosocomial transmissions from HWs to patients, to detect fresh LTBI in HWs, and to treat them if necessary [10]. Occupational medical check-ups are governed by the German Ordinance on Occupational Health Care (Verordnung zur arbeitsmedizinischen Vorsorge, *ArbMedVV*) [11]. It differentiates between mandatory and voluntary check-ups. For workers in wards involving infectious diseases, in particular tuberculosis, for workers in pulmonology where contact with TB patients is frequent, and for workers in laboratories where testing is conducted for *Mycobacterium tuberculosis* (M. Tb), regular testing is mandatory. In the case of mandatory testing, employees must regularly visit an occupational physician.

A consultation with the physician may be sufficient and the worker can then decide whether and to what extent an examination should be conducted. This option was introduced when the Occupational Medical Check-Up Regulation was amended in 2013 [12]. All other workers are offered a check-up after incidental contact with an infectious patient. It is equivalent to the contact tracing investigations performed on contact persons of patients suffering from infectious TB by the Public Health Authority.

Comparison figures on LTBI prevalence among the general population are unavailable, as testing is only performed in cases where persons have had close contact in an outbreak. No routine screening is performed. Comparison groups, therefore, often consist of employees without direct patient contact but from the same setting as, for example, administrative staff within the hospital [13]. In a dissertation currently in progress, non-health workers (non-HWs) in Germany were examined for LTBI for the first time [14].

The purpose of this study is to examine whether HWs who have routine contact with TB or work on wards and in departments where accidental TB exposure occurs are at greater risk of LTBI than employees in other sectors (viz. non-HWs) who are not subject to occupational LTBI infection risk.

## 2. Materials and Methods

### 2.1. Study Design and Subject

This cross-sectional study aimed to assess the additional risk of HWs for LTBI. The interferon-gamma release assay testing (IGRA) is used to detect the presence of LTBI or TB, respectively.

### 2.2. Data Source

This study is based on two opportunity samples. The first sample was from a group of workers in Hamburg who had no apparent occupational connection with the health care system but who received IGRA testing in 2018 and 2019 in connection with a doctoral study. This control group was recruited as an occasional sample through a partnership with the company physicians of the Hanseatic Center for Occupational Medicine (*Hanseatisches Zentrum für Arbeitsmedizin GbR*) and is thus not representative of the German population. Criteria for inclusion were an employment contract in a non-medical profession, a minimum age of 18 and a maximum age of 67.

The non-HW group consisted of employees of the following occupational groups: education, office work, chemical industry, service sector (e.g., hairdresser) and participants who trained for a profession or were still in training for an unspecified profession outside the health sector. The group of HWs consisted of nurses, physicians, administration staff, technicians, trainees, therapists and others. The workplaces were divided in internal medicine, admission ward, infection ward, geriatric care, radiology, laboratory, pathology, intensive care unit, administration and other clinical wards.

The second sample consists of workers in the health care sector (hospitals, nursing homes and outpatient care units). This data was collected between 2006 and 2017 using a questionnaire and an IGRA. The HWs to be tested by the occupational health specialists throughout Germany were selected in accordance with the Ordinance on Occupational Health Care [12]. Members of the “company physicians’ network” provided the data in anonymous form for the study center in Hamburg.

All HWs who had routine contact with TB patients, regardless of whether they were protected or not upon contact, were examined at intervals ranging from once a year to every third year, depending on how the appointed physician assesses the risk. In addition, after accidental TB exposure, all HWs of wards and departments where TB patients are not usually treated are offered a voluntary check-up [15].

### 2.3. Variables

First, the non-HW sample and the HW sample were descriptively studied for positive test results and presented in tabular form. Then, the sample was reduced to eliminate those persons born in high-risk countries (more than 40 TB cases per 100,000 inhabitants per year). An analysis of persons born in countries at high risk of TB is presented in the supplement. We decided on this approach because we found an unusually high positive test result rate of 67% among the three subjects born in high-risk countries in the non-HW sample. The presentation of both results, (i) without and (ii) with the subjects born in high-risk countries in the supplement allows for a type of sensitivity analysis using the presentation of both estimates.

Variables present in both samples (age, gender, past TB history, country of birth [Germany]) were used as independent variables for PS matching.

### 2.4. Data Collection

The subjects from the two groups were asked to complete a questionnaire after providing a declaration of consent. The questionnaire included both sociodemographic details—age, gender, country of birth, and years spent living in Germany—and aspects related to their past TB history (past TB infections, participation in contact studies, TB infection in family/among close friends, occupational exposure, etc.). In the non-HW group, subjects were also asked about immunosuppression and about any volunteer activities in medical fields they may be engaged in to exclude potential confounders.

### 2.5. Test Method

The subjects in both the non-HW and HW groups had blood samples taken to determine by QuantiFERON^®^ test (QFT) whether the subject had a latent or active tuberculosis infection. The blood samples were taken by their respective company physicians. At least 5 mL of blood was drawn from the subjects in a lithium heparin tube. The tubes were stored as per manufacturer specifications (www.QuantiFERON.com) at room temperature (22 ± 5 °C) and transported to the laboratory by a courier within no more than 8 h.

In the laboratory, 1 mL of blood was transferred from the sample in the lithium heparin tube into each of four test tubes and briefly shaken to achieve adequate homogeneity. The first tube, *QuantiFERON Nil,* was used as a negative control. Tubes 2 and 3 were coated with the *M. tuberculosis*-specific antigens ESAT 6 and CFP-10. Tube 4 contained mitogen and was used as a positive control. After 16 h of sample incubation at 37 °C, the samples were centrifuged for 15 min. The samples then underwent an enzyme-linked immunosorbent assay (ELISA) and were analyzed using the manufacturer’s software. The interferon-gamma (INF-γ) values in tubes 2 and 3 minus the negative control (tube 1) were read. In the HW sample, most tests were performed with an earlier version of the QFT containing ESAT 6 antigens only.

A test was deemed to be positive if the tested INF-γ value in one of the two antigen tubes was at least 0.35 IU/mL after deduction of the zero control. If both values were under 0.35 IU/mL, the result was deemed to be negative. For a test result to be conclusive, the positive control must have had an IFN-γ concentration of ≥0.5 IU/mL after deduction of the nil control (mitogen nil value); otherwise, the QFT was deemed inconclusive.

### 2.6. Handling of Positive-Testing Subjects

All subjects in the non-HW group were able to decide for themselves beforehand whether they wished to be notified of their test result. Notification of test results was sent by post. Subjects who tested positive were invited to a consultation with their company physician. During the consultation, subjects were advised that the QFT Plus test only provides indications of a latent or active tuberculosis infection. Further diagnostic measures were also discussed based on the individual risk profile, and the subjects were referred to a pneumology specialist.

In the HW group, care was provided by the responsible company physician following the recommendations for occupational medical consultations and examinations following TB contact based on the recommendations of the German Central Committee against Tuberculosis [16]. All participants with a positive IGRA at baseline or showing a conversion were offered a clinical and radiological examination to rule out active TB.

LTBI is defined as a positive IGRA result in the absence of medical symptoms and signs of an active TB in the chest X-ray. Active TB was not detected in the non-HW group or in the HW group.

### 2.7. Statistical Analysis

Descriptive analyses were performed for categorical variables with case figures and relative frequencies and the groups were compared using Fisher’s exact test where appropriate. Continuous values were analyzed using averages, presented in tabular form and compared using *t*-tests. Where there was no normal distribution, Wilcoxon’s rank-sum test was applied for group comparison. The propensity score was determined using a binary logistic regression model with correction using the Firth method [17,18]. Group membership in “health workers” vs. “non-health workers” was used as the dependent variable here. In choosing the independent variables, we limited ourselves to key risk factors for LTBI present in both samples. A binary logistic regression with a positive IGRA test as a target value was also used to study factors of influence on the prevalence of LTBI.

Each individual study subject in the non-HW sample was then assigned to a group of ten persons from the company physicians network sample using the “PS matching” method (1:10 matching) [19].

The nearest-neighbor method was applied (i.e., each was paired with the partner nearest in terms of PS). This allows comparable groups to be formed with regard to the aforementioned variables—similarly to a randomized study, which is presently the gold standard for comparative studies [20,21].

Risk factors for LTBI were analyzed using a multivariate logistic regression model. All two-way interactions were examined and remained in the model when statistically significant.

The statistical analysis program R version 4.0.1 [22] was used for the statistical analyses. The significance level was set at 5%.

### 2.8. Ethics Vote

Professional legal advice for this study was obtained from the Hamburg Ethics Committee (Reg. No. PV5713). All subjects submitted a written declaration of consent prior to their participation.

## 3. Results

The HW data set contains information on 4882 persons—of which, 30.7% were examined in environmental investigations after contact with a TB-infected person. The elimination of the subjects born in high-risk countries resulted in a reduction in their number of 325 (6.7%) in the HW group to 4557 and of 3 (2.9%) in the non-HW group to 100. From the more than 40-fold larger data set, a comparable group could be drawn this way, which was 10-fold larger due to the matching ratio applied.

**Figure 1:**
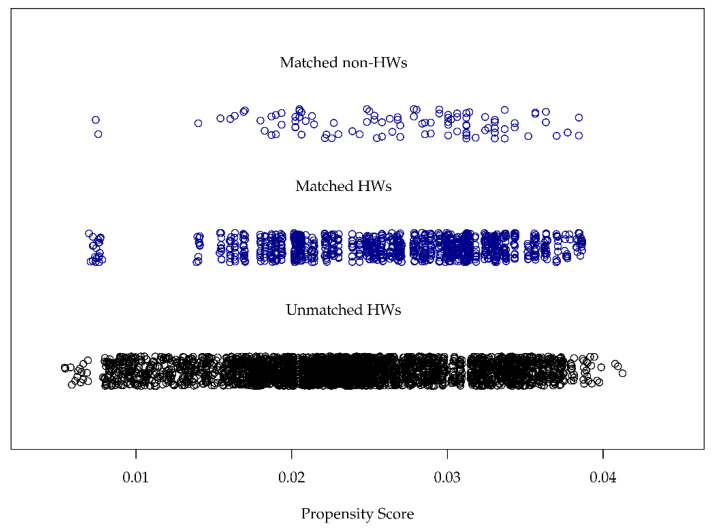
The propensity scores (PS) of the populations: at the top, the non-HWs were each matched with 10 suitable HWs. The lower point cloud shows the PS for the unmatched subjects from the HW population.

PS matching enabled the right partner in terms of the propensity score to be found (see Figure 1). Particularly in terms of the ages of the subjects in the total HW sample, there were significant differences between the groups (see Table 1). The country of birth for 17% of the subjects among HWs was not Germany; in the comparison group, it was 11%. PS matching provided consistently balanced conditions (see Table 2) in terms of the variables used.

**Figure 2:**
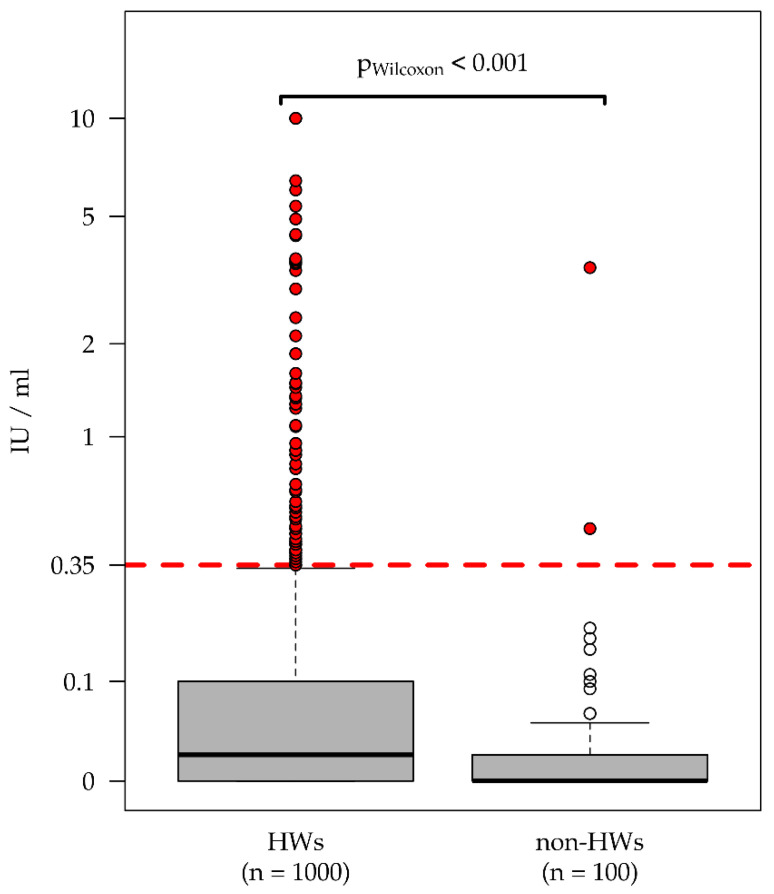
Amount of INF-γ value (tube TB1) by group (HWs vs. non-HWs).

A comparison of the collected IGRA values reveals that HWs with possible occupational contact to patients with TB infection tend to exhibit higher values than the non-HW comparison group. When reviewing the distribution of the test results displayed in Figure 2, 7.2% of the HW group (72/1000) and 2.0% of the non-HW group (2/100) had a positive IGRA test result. In a univariate analysis, this corresponds to an odds ratio (OR) of 3.86 (95% CI: 0.99 to 32.5; *p* = 0.056) for an increased likelihood of a positive test result in the HW group.

**Figure 3:**
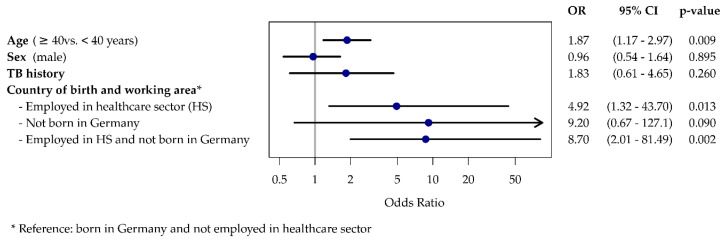
Forest plot for the results of the logistic regression model with IGRA test positive as the dependent variable. The variables age, sex, TB history and all four possible combinations of country of birth and employment as HWs are depicted as independent variables.

The multivariate analysis, taking into account age groups, gender, TB history, country of birth (Germany vs. not Germany) and employment in the health care sector, gives the following results: we identify an age effect where the risk increases as the age group increases, which is statistically significant (*p* = 0.009). There is no evident gender effect (*p* = 0.89). A near doubling of the risk for subjects with a TB history was demonstrated, and there was an effect from both country of birth (OR = 9.2; 95% CI: 0.67 to 127.1; *p* = 0.090) and from employment in the health care sector (OR = 4.9; 95% CI: (1.32 to 43.7); *p* = 0.013). However, the latter two effects combined with each other do not have a multiplicative effect, but result in an OR of 8.7 (95% CI: 2.0 to 81.5; *p* = 0.002) (Figure 3) when interacting with one another.

In total, 8% of the non-HW group (8/100) consisted of subjects not born in Germany; 25% of this subgroup (2/8) returned positive test results. For countries of birth with incidence rates of more than 40 tuberculosis cases annually per 100,000 inhabitants, the proportion testing positive was 67% (2/3). In the HW group, the proportion in this subgroup was 16% (52/325). This random, yet unusually high, discrepancy means that an analysis of subjects born in high-risk countries shows a more extreme situation relating to the factor of influence “foreign born”.

Taking those born in high-risk countries into account, the estimator for the OR among “foreign born” is 18.9 (95% CI: 2.6 to 217.1; *p* = 0.004), while for HWs not born in Germany the OR decreased to 7.62 (95% CI: 1.8 to 70.3; *p* = 0.003) (see Supplement Figure S2).

## 4. Discussion

This is the first study comparing the LTBI risk of HWs with a non-exposed group outside the health care sector. We observed a prevalence of 2.0% for non-HWs and 7.2% for HWs. Due to professional exposure, there has long been a scientific interest in analyzing LTBI among HWs. In a review, Peters et al. studied the occupational risk of LTBI among medical personnel in low-incidence countries and found prevalence rates among HWs from 0.9% to 85.5% [1]. The values found in this study lie within the range of these widely distributed and regionally varying prevalences.

### 4.1. Occupation as a Risk Factor

In their work on LTBI among HW, Schablon et al. [15] found that workers in areas that inherently entailed a risk of exposure to TB (e.g., infectious disease wards or pathology) were at greater risk of LTBI. Bukhary et al. [23] found in their studies of HWs that medical personnel were at greater risk of LTBI. Torres Costa [24] also described an increased risk of LTBI among physicians. Other studies involving HWs also described a higher risk for physicians and nursing staff [25,26].

On the other side, Schablon et al. [15] also found an astonishingly high rate of positive IGRA test results (14.2%) among subjects whose occupational group was summarized as “administrative”. Peters et al. [1] also concluded that an “administrative” working area, comprising a heterogeneous group of employees in reception or management, increases the occupational risk of LTBI. For medical or nursing personnel, on the other hand, they identified no increased occupational risk of LTBI.

### 4.2. Comparison of HWs and Non-HWs

In this study, we found a higher LTBI prevalence among HWs and higher IGRA values where testing was positive. Lower IGRA values, especially test results in the borderline zone up to a level of 0.7 IU/mL, have a higher probability of reversion [27].

Baussono et al. [28] compared LTBI prevalence rates among HWs based on tuberculin skin tests with those of the general population in low-incidence countries. They also found a higher LTBI risk among HWs. In their meta-analysis, Uden et al. [13] compared HWs who had direct patient contact with a control group comprising employees of a hospital with no patient contact and people in the general population. They too demonstrated a higher prevalence of LTBI (37%) and active TB among HWs and concluded on this basis that the overall risk for LTBI among HWs remained significantly higher than the risk for the general population.

### 4.3. Risk Factors

In addition to examining prevalence rates and occupational risk of LTBI among HWs, these studies also identified other independent risk factors for LTBI. Schablon et al. [15] described an age of over 55 and a prior TB infection in the history of the subject as risk factors for a positive QFT result.

Other studies also demonstrated that increased age resulted in greater risk of LTBI [26,29,30]. Furthermore, the duration of employment in health care appears to be a risk factor for LTBI among HWs [31,32].

Another quoted risk factor for LTBI in scientific work concerning LTBI among HWs is a migrant background, which may not necessarily be related to birth but also to travel or business activities in high-risk countries [15,29]. For subjects with a migrant background from the non-HW group, we found an odds ratio of 9.2. Subjects not born in Germany who were employed in health care, on the other hand, had an odds ratio of 8.7. While the estimators do not differ from each other in a statistically significant fashion (reflected in the heavy overlap in confidence intervals), this may provide a hint of the additional caution that workers in health care tend to apply. Specifically HWs use masks, gloves and gowns for protection. Patients with active TB are isolated and special ventilation systems are available. We suspect that HWs are more likely to adhere to the general precautions (i.e., safe distance and breathing mask) that should be observed when dealing with potentially infectious persons.

### 4.4. Limitations

The number of cases in the non-HW comparison group is small. The comparison group comes from a different context (both in terms of time and of environment), meaning that the derived figures can only serve as a rough estimate, and should not be reported separately from the study design. Specifically, the non-HW sample was between 18 and 67 years old, which excludes the share of the older population. Furthermore, the gender distribution in the non-HW group was not representative for the German population, as the majority of the participants in the sample were female (79%). Concerning the representativeness of the sample in terms of geographic location, we have a higher incidence rate of 9.7 cases per 100,000 inhabitants in Hamburg than in Germany overall (6.5 cases/100,000 inhabitants) [6]. The age effect is, as was to be expected from prior knowledge, associated with increased age entailing greater risk. In the studies by Knierer et al. [33] and Gallegos et al. [34], relatively high prevalence rates were also found among young subjects, but this was probably due to their originating from countries with higher incidence rates. Prevalence rates from non-exposed groups would be of interest to estimate the risk in the general population.

## 5. Conclusions

This study shows that working in the health care sector entails a risk of TB infection. Despite the declining tuberculosis incidence rates in Germany, a comparison with non-exposed professional groups shows that HWs are at greater risk of LTBI. Therefore, preventive medical check-ups still seem to be indicated.

Furthermore, this study showed that HWs have both a higher risk for LTBI and higher IGRA values. It would be advisable for the individuals in this group to identify recent or current infections and preventive treatment should be recommended to reduce the risk of progression to active TB.

## Figures and Tables

**Table 1:** Group comparison of available subjects prior to matching.

	HW (*n* = 4882)	Non-HW (*n* = 103)	*p*-Value
Age (mean (SD)) in years	38.6 (11.9)	35.7 (10.8)	0.013
Sex (male)	1117 (22.9%)	23 (22.3%)	1.000
TB history	333 (6.8%)	4 (3.9%)	0.320
Country of birth (other than Germany)	734 (17.1%)	11 (10.7%)	0.264
Positive IGRA test (≥0.35 IU/mL)	376 (7.7%)	4 (3.9%)	0.188

**Table 2:** Group comparison excluding subjects whose country of birth is a TB high-risk country after PS matching 1:10.

	HW (*n* = 1000)	Non-HW (*n* = 100)	*p*-Value
Age (mean (SD))	35.0 (11.1)	35.6 (10.9)	0.620
Sex (male)	227 (22.7%)	22 (22.0%)	1.000
TB history	35 (3.5%)	3 (3.0%)	1.000
Country of birth (other than Germany)	93 (9.3%)	8 (8.0%)	0.856
Positive IGRA test (≥0.35 IU/mL)	72 (7.2%)	2 (2.0%)	0.056

## Supplementary Materials

The following are available online at https://www.mdpi.com/1660-4601/17/13/4643/s1. The results taking into account the subjects from TB high-risk countries are reported in Figure S1: Box plot including subjects born in a TB high-risk country after PS matching 1:10; Figure S2: Forest plot including subjects born in a TB high-risk country. Table S1: Group comparison including subjects born in a TB high-risk country after PS matching 1:10.
