# Supplementary material for: Risk Analysis of Latent Tuberculosis Infection among Health Workers Compared to Employees in Other Sectors
Source: Int J Environ Res Public Health. 2020 Jun 28;17(13):4643. doi: 10.3390/ijerph17134643 (PMC7370114; doi:10.3390/ijerph17134643)
Supplement: Supplementary file 1 [file ijerph-17-04643-s001.pdf]

Supplement

Table S1. Group comparison including subjects born in a TB high-risk country after PS matching 1:10.

	**HW** (n = 1030)	**non-HW** (n = 103)	**p-value**
Age (mean (SD))	35.1 (10.6)	35.7 (10.8)	0.583
Sex (male)	223 (21.7%)	23 (22.3%)	0.900
TB history	44 (4.3%)	4 (3.9%)	1.000
Country of birth (other than Germany)	130 (12.6%)	11 (10.7%)	0.641
Positive IGRA test (≥ 0.35 IU/ml)	74 (7.2%)	4 (3.9%)	0.304

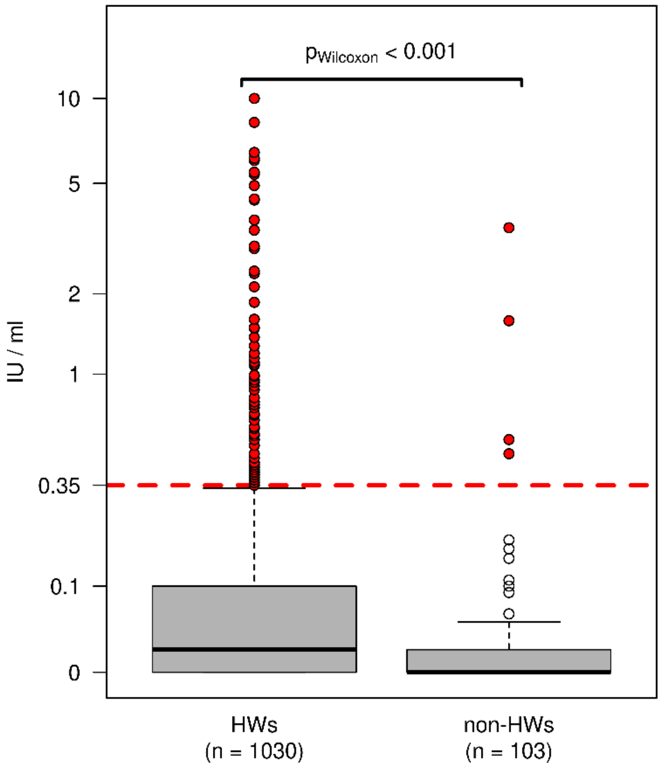

Figure S1. Amount of INFγ-value (tube TB1) by group including subjects born in a TB high-risk country (HW vs. non-HW)

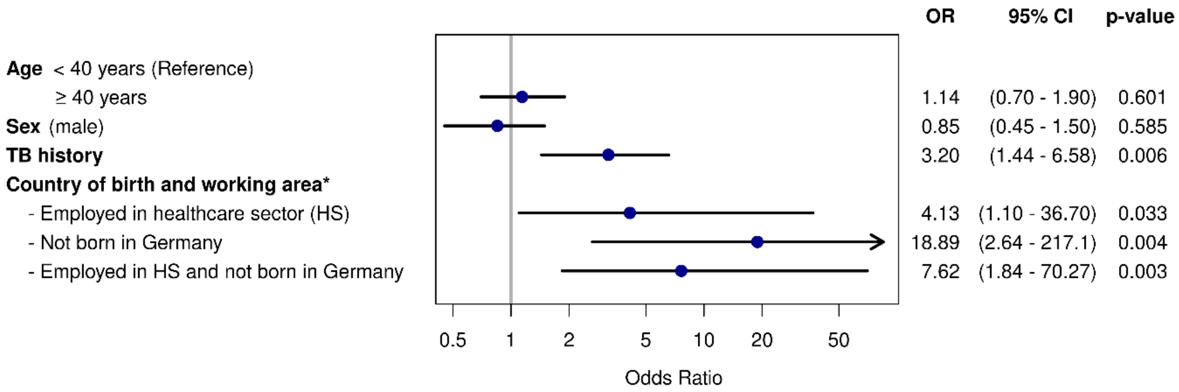

* Reference: born in Germany and not employed in healthcare sector

Figure S2. Forest plot for the results of the logistic regression model with IGRA test positive as the dependent variable. The variables age, sex, TB history and all four possible combinations of country of birth (including subjects born in a TB high-risk country) and employment as HW are depicted as independent variables.
